# Adénocarcinome sur une exstrophie de vessie chez un patient adulte: à propos d’un cas

**DOI:** 10.11604/pamj.2018.29.197.15291

**Published:** 2018-04-03

**Authors:** Bounoual Mohammed, Omana Jean Paul, Ahsaini Mustapha, Mellas Soufiane, El Ammari Jalaleddine, Tazi Mohammed Fadl, El Fassi Mohammed Jamal, Farih Moulay Hassan

**Affiliations:** 1 Service d’Urologie, CHU Hassan II de Fès, Maroc

**Keywords:** Exstrophie vésicale, adénocarcinome, cystectomie radicale, Bladder exstrophy, adenocarcinoma, radical cystectomy

## Abstract

L'exstrophie de vessie est une pathologie malformative rare avec une incidence d'un cas pour 50000 naissances. Non traité à temps expose à deux principales complications: l'insuffisance rénale et La cancérisation de la plaque vésicale avec un risque allant jusqu'à 200 fois la normale, qui survient généralement vers la quatrième et la cinquième décennie. Dans 95% il s'agit d'un adénocarcinome et 5% un carcinome épidermoïdes. Nous présentons un cas rare d'adénocarcinome développé sur une exstrophie vésicale chez un patient de 61 ans qui a subi d'une exérèse de la plaque vésicale emportant toute la masse tumorale avec une dérivation urinaire non continente type bricker.

## Abstract (en)

Bladder exstrophy is rare malformation pathology with an incidence of about 1 in 50,000 newborns. Not treated in time exposes to two main complications: kidney failure and bladder plaque cancer with a risk up to 200 times normal, which usually occurs around the fourth and fifth decade. In 95% it is adenocarcinoma and 5% squamous cell carcinoma. We present a rare case of adenocarcinoma developed on a bladder exstrophy in a 61-year-old patient who underwent an excision of the bladder plate carrying the whole tumor mass with a non-continent urinary diversion type bricker.

## Introduction

L'exstrophie de vessie est une malformation congénitale complexe qui se caractérise par l'absence de toute la paroi abdominale antérieure médiane sous ombilicale et de la paroi antérieure de la vessie, touchant aussi l'urètre, la ceinture pelvienne, le périnée et les organes génitaux externe. Elle se présente comme une plaque rouge, vultueuse, qui bombe sous la poussée abdominale et qui fait intégralement partie de cette paroi appelée la plaque vésicale associé à un épispadias et une diastasis de la symphyse pubienne [1-4]. Une exstrophie vésicale se reconnaît dés le 1^er^ examen d'un nouveau-né, le tableau est dominé par l'incontinence urinaire, il s'y ajoute des douleurs, une irritation cutanée et vésicale [3]. Le traitement chirurgical de l'exstrophie de vessie est complexe, comportant plusieurs méthodes chirurgicales consistant sur la réparation vésicale, la dérivation des urines, la réparation pariétale et celle des organes génitaux externes [5]. La prise en charge doit être précoce aux premiers mois de vie pour avoir un meilleur résultat fonctionnel et esthétique [1, 4, 5]. Une exstrophie vésicale négligée expose aux deux principaux risques: Le retentissement sur le haut appareil par infection ascendante et sténose due à la fibrose et La cancérisation [1, 2, 6, 7]. Nous rapportons un cas d'un patient avec une exstrophie vésicale non traitée compliquée d'un adénocarcinome.

## Patient et observation

**Figure 1:**
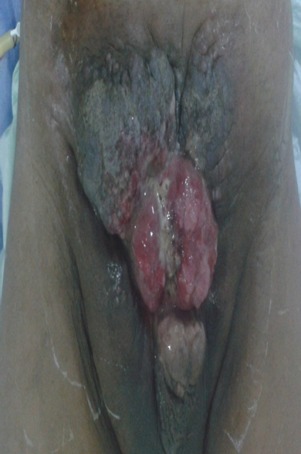
Image montrant un patient avec un adénocarcinome sur exstrophie vésicale complexe

**Figure 2:**
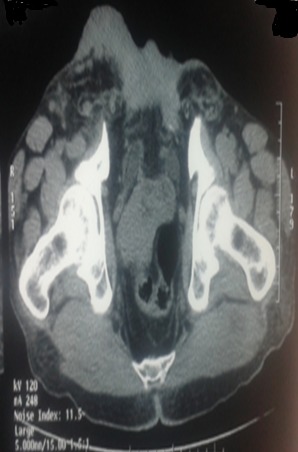
TDM abdominopelvienne en coupe transversale montrant la masse tumorale implantée sur la plaque vésicale

**Figure 3:**
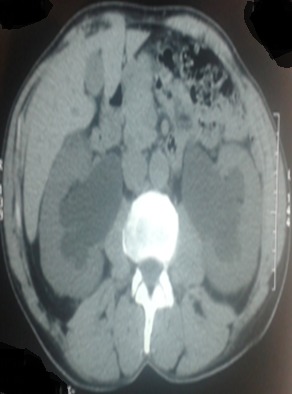
TDM abdominale en coupe transversale montrant la dilatation urétéropyélocalicielle

**Figure 4:**
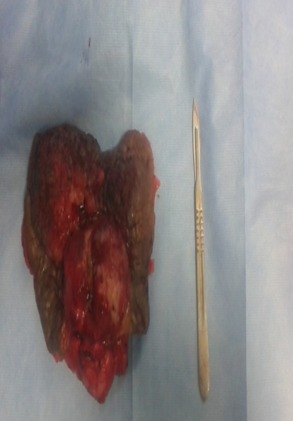
Pièce opératoire (cystectomie radicale)

**Figure 5:**
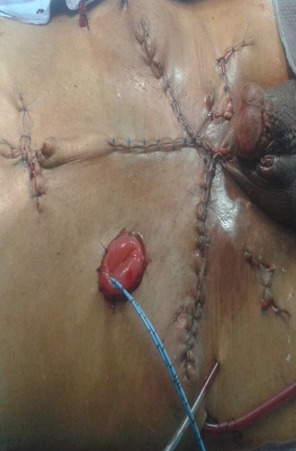
Image post opératoire montrant la qualité de la fermeture pariétale

Il s'agit d'un patient de 61 ans, célibataire, qui présente une exstrophie vésicale depuis la naissance jamais traité, consulte pour l'apparition d'une masse hypogastrique avec des lombalgies bilatérales. L'examen clinique avait montré une complexe exstrophie vésicale épispadias classique avec une masse surinfecté de 6 cm environ au dépend de la plaque vesicale (Figure 1). Une biopsie de cette masse a objectivé un adénocarcinome moyennement différencié de type lieberkinien. Un bilan para-clinique avait objectivé une insuffisance rénale avec une créatinine à 57mg/ml. Une TDM abdomino-pelvienne (Figure 2, Figure 3) avait objectivé une classique complexe exstrophie vésicale épispadias et diastasis pubienne avec une masse tumorale au dépend de la plaque vésicale envahissant les deux méats urétéraux et déterminant une UHN bilatérale, pas d'atteinte ganglionnaire pelvienne ni métastase à distance. Le patient fut admis au bloc, nous avons réalisé une ablation de la plaque vésicale emportant toute la masse tumorale (Figure 4), avec une dérivation urinaire externe: urétérostomie cutanée trans-iléale type bricker. La fermeture de la paroi abdominale antérieure était difficile vue la perte de substance cutaneo-aponévrotique (Figure 5). Les suites opératoires étaient simples. L'étude anatomopathologique de la pièce opératoire montrant un adénocarcinome moyennement différencié de type lieberkinien infiltrant le muscle vesicale classé pT2, le curage ganglionnaire ilio-obturateur ne montrant pas d'atteinte ganglionnaire. Le suivi de patient a été marqué par la normalisation de sa fonction rénale. Il se porte bien sans aucune preuve de récidive à ce jour.

## Discussion

L'exstrophie vésicale est une pathologie malformative très rare liée à l'échec du développement urogénital au début de la gestation secondaire à une partition anormale de la membrane cloacale [2]. Le diagnostic de l'exstrophie vésicale pourrait se faire en période prénatale avec les progrès en imagerie obstétricale [2,6]. La prise en charge devrait être réalisée dans la période néonatale ainsi on prévient les complications qu'elle encourt que ce soit sur le plan psychosocial et sexuel et aussi un traitement plus aisé permettant de respecter l'image corporelle en insistant sur la fermeture vésicale et abdominale ainsi la réparation cervico-sphincterienne, la réparation de l'épispadias et la reconstruction fonctionnelle et esthétique des OGE ce qui assure une bonne continence urinaire, un bon fonctionnement sexuel et une protection de la fonction rénale [1,4,5]. Devant des conditions socio économiques défavorables, on pourra se rencontrer avec des cas d'exstrophie vésicale dite négligée qui ne consultait qu'à un âge avancé c'est le cas de notre patient. Le retard de prise en charge expose à la dégradation de la fonction rénale par les phénomènes inflammatoires et dégénératifs de la muqueuse vesicale obstruant les méats urétéraux et à un potentiel malin de la muqueuse vésicale exstrophiée suite à l'irritation chronique et l'infection conduisant à une transformation métaplasique de l'urothélium. L'adénocarcinome est le type histologique le plus commun dans les cas d'exstrophie de la vessie, ce qui représente 95% des cas, alors que le carcinome épidermoïdes ne représente que 3% à 5% des cas [1,2,6,7]. La prise en charge de l'exstrophie vésicale chez l'adulte est délicate consiste sur l'exérèse totale de la plaque avec réalisation d'une dérivation urinaire continente notamment la poche iléocæcale continente de Benchekroun ou dérivation non continente comme l'urétérostomie cutanée trans-iléale type bricker [1,3,6,7], c'est le cas de notre patient. La chirurgie reconstructrice de la vessie est déconseillée chez l'adulte vue le risque important d'échec et de cancérisation de la plaque [2]. La fermeture pariétale pose un problème car la réparation sous la tension causée par le diastasis de la symphyse pubienne et la perte de substance peut conduire à la déhiscence de la plaie ainsi la cicatrisation est inférieur dans de tels cas. L'utilisation de la maille ou l'utilisation d'un lambeau du fascia lata du tenseur peut permettre une fermeture pariétale sans tension [1,6]. L'ostéotomie chez l'adulte est généralement non faite vue le risque d'instabilité osseuse de bassin [1]. Dans notre cas, parce que le défaut était plus petit, il était couvert en mobilisant la gaine des deux côtés, et la couverture de la peau a été faite avec l'aide des collègues de chirurgie plastique. Devant un adénocarcinome ou carcinome épidermoïdes sur vessie exstrophique, la cystectomie avec curage ganglionnaire pelvien étendu constitue le seul traitement curatif au stade localisé [1,2,6,7]. La radio chimiothérapie est réservée au stade localement avancé ou métastatique ou chez les patients qui refusent la chirurgie [1,2]. Le pronostic de cette entité histologique est en général réservée vue l'agressivité qui présente [1]. Le suivi de ces patients est similaire à celui d'un carcinome urothéliale consistant sur un examen clinique, une imagerie de contrôle et une fonction rénale pour dépister une éventuelle insuffisance rénale [1,2].

## Conclusion

L'exstrophie vésicale chez l'adulte est une véritable tragédie sexuelle, psychologique et sociale. La PEC précoce permet de construire un appareil urinaire et génital aussi proche de la normale que possible. L'exstrophie négligée expose à l'altération de la fonction rénale et à un risque fois 200 d'avoir un adénocarcinome dont le traitement est la cystectomie totale avec dérivation urinaire continente ou non.

## Conflits d’intérêts

Les auteurs ne déclarent aucuns conflit d'intérêts.
